# 

**Published:** 1814-08

**Authors:** 


### MONTHLY CATALOGUE OF MEDICAL BOOKS.

AN INDEX to the Anatomical, Medical, Chirurgical, and Physiological Papers contained in the Transactions of the Royal Society of London, from the commencement of that work to the end of the Year 1813; chronologically and alphabetically arranged. 4to. boards, 10s. 6d.

Professor Davy's interesting Inquiries concerning the Relation of Galvanism to Living Action; illustrated in the Removal of Topical Complaints by the Application of Simple Galvanic Circles: and recommended as an useful Assistant Branch of Medicine. By Matthew Yatman, Esq. 8vo. sewed, 2s.

A Manual of Mineralogy. By Arthur Aikin, Secretary to the Geological Society. 12mo. boards, 5s.

---

### *TO THE READERS OF THE MEDICAL JOURNAL.*

PEACE, *for which we have sighed so many years, has at length revisited Europe with healing in its wings, and enables the* MEDICAL AND PHYSICAL JOURNAL *more widely to diffuse its advantages by a greatly-extended circulation.*

*We have thus the gratification to announce, that whilst contributing to the extension and improvement of medical knowledge and general science, the value of our labours rises every month.*

*The* MEDICAL AND PHYSICAL JOURNAL *now circulates as the* GAZETTE OF THE FACULTY *in all the States of* EUROPE, *the whole of civilized* AMERICA, AFRICA, *the* EAST *and* WEST INDIES, *and indeed in every enlightened portion of the Globe.*

*This pre-eminence enables us to obtain and convey information of the first importance to the Profession throughout the World.*

*The state of Medical Literature abroad will be an early and very prominent character of our Continental Advantages.*

*Lists will be subjoined from time to time of new Foreign Works in Medicine, Pharmacy, Anatomy, Surgery, Chemistry, Natural History, and the other Branches of Philosophy connected with the Profession, as soon as they appear.*

*In short our future pages, in every department, will testify that our attention to the interests of our most numerous and respectable friends and correspondents increases with their daily increasing numbers, and will amply repay the patronage by which this Journal is distinguished.*

*August* 1, 1814.

